# Retromer enhancement rescues endosomal recycling defects induced by SORL1 deficiency

**DOI:** 10.1002/alz.084838

**Published:** 2025-01-03

**Authors:** Swati Mishra, Charles A Williams, Jessica E. Young

**Affiliations:** ^1^ University of Washington, Seattle, WA USA

## Abstract

**Background:**

The *SORL1* gene (SORLA) is strongly associated with risk of developing Alzheimer’s disease (AD). SORLA is a regulator of endosomal trafficking in neurons and interacts with retromer, a complex that is a “master conductor” of endosomal trafficking. Because of its size, SORLA is difficult to target therapeutically. In this study, we used small molecules that enhance retromer expression to rescue cellular phenotypes induced by SORLA deficiency or by SORLA missense variants in hiPSC‐derived cortical neurons and microglia.

**Methods:**

We treated SORLA KO, SORLA ± and SORLA variant (SORLA^Var^) neurons and microglia with TPT‐260, a small molecule that enhances retromer expression and function. We tested the effects of TPT‐260 on endosome size, SORLA‐retromer cargo localization, Amyloid beta secretion, phospho‐tau expression in neurons and phagocytosis and cytokine release.

**Results:**

We observed that TPT‐260 significantly induced expression of retromer subunits in our cells. In neurons, TPT‐260 treatment rescued endosome swelling and abnormal SORL1‐cargo localization. In addition, we observe significant reductions in Amyloid‐beta secretion and intracellular phospho‐Tau. Interestingly, rescue of these phenotypes was dependent on whether at least one functional copy of SORLA was expressed and on which AD‐associated variant was present. Evaluation of phenotypes in microglia are currently in progress.

**Conclusion:**

Our work shows that treatment with a small molecule that increases retromer expression *in vitro* and enhances retromer‐related trafficking can reduce important cellular and neuropathologic phenotypes in human AD neuronal models. Using TPT‐260, we broadly show that enhancement of retromer‐related trafficking can fully or partially rescue deficits induced by loss of *SORL1*. This study builds on previous work that has used these and other retromer‐targeting small molecules in animals and human cells, suggesting that studies such as this could represent an important preclinical step in identifying new therapeutic molecules for AD.

